# Microbial ecology of sandflies—the correlation between nutrition, *Phlebotomus papatasi* sandfly development and microbiome

**DOI:** 10.3389/fvets.2024.1522917

**Published:** 2025-01-22

**Authors:** Slavica Vaselek, Bulent Alten

**Affiliations:** Department of Biology, Faculty of Science, Hacettepe University, Ankara, Türkiye

**Keywords:** sandfly, *Phlebotomus*, bacteria, microbiome, nutrition, development

## Abstract

The role and the impact of the microbial component on the biology, ecology, and development of sandflies is largely unknown. We evaluated the impact of larval nutrition on laboratory-reared sandflies in correlation to the abundance of food, light starvation, and food with/without live microbiome, by monitoring the survival and development of immature stages, and the longevity of adult sandflies. Within this study we examined 360 larvae, 116 pupae, and 120 adult flies of *Phlebotomus papatasi* for the microbial gut content. The data showed that the presence of a live and diverse microbiome plays a role in the development and survival of larvae. The mortality rate of the larvae was higher, and larval development was longer for sandflies maintained on microbiome-depleted medium, in comparison to the larvae fed with medium containing alive and complex microbiome. Actively feeding larvae reduce microbial abundance and diversity of the medium. The microbial content of the larval gut depends on the composition of the rearing medium, indicating a potential attraction to certain bacteria. The microbial content of the pupa gut was severely diminished, with overall survival of two bacterial species in adult insects - *Ochrobactrum intermedium* (found in 95% of dissected adults) and *Bacillus subtilis* (16%). Further microbial studies may aid in developing biological control methods for sandfly larval or adult stages.

## 1 Introduction

Phlebotomine sandflies (Diptera, Psychodidae) are hematophagous insects of great medical and veterinary importance on a global scale (1). Sandflies are able to transmit protozoan, bacterial, and viral pathogens (2), among which protozoan parasites from the genus *Leishmania* (Kinetoplastida, Trypanosomatidae) — the main causative agents of leishmaniasis, are of particular importance to veterinarians and physicians around the world. Leishmaniasis is a severely underreported disease, counting 1–2 million reported cases annually, with serious implications that incidence numbers are significantly higher (3, 4). Despite the increasing number of cases and continuous disease spread, there are no approved human vaccines, while currently used therapeutic approaches and control strategies show unsatisfactory results (5–8).

Major health authorities around the world persistently emphasize the urgent need for the development of new leishmaniasis control methods. In order to develop effective leishmaniasis control measures, among others, it is necessary to understand sandfly vectorial potential, biology, and ecology (9). A comprehensive understanding of sandfly biology requires that sandflies be studied in an ecological context, with the microbiome as an important component of the system. In the past, the microbial component and its impacts on sandfly life, fitness, fecundity, vectorial potential, etc. were greatly overlooked. The recent resurgence of interest in sandfly microbial community investigations was prompted by abundant evidence highlighting the role of microbes in the control of vector-borne diseases such as malaria, dengue, Zika, Chagas disease, etc. (10–12).

Sandflies inhabit various environments, providing them with ample opportunity to encounter and interact with diverse microbes from the environment. Their complex life cycle, coupled with the distinct lifestyle requirements and dietary preferences of immature and adult stages (e.g., larvae that feed on decomposing organic material versus adult flies that feed on the plant juices, blood, etc. (13)) supports a maintenance of a rich gut microbiome that can be inherited and acquired (14, 15). As *Leishmania* development in the sandfly is confined to the digestive tract, the parasites will inevitably encounter and interact with this diverse microbiome (16). The gut microbiome of sandflies can either hinder or facilitate the survival and development of *Leishmania* within the sandfly (17, 18). Moreover, the microbiome egested during sandfly feeding significantly contributes to host infection by inflammasome activation (19). Up to this point, only a limited number of studies focused on investigations of the microbial impact on sandfly biology and ecology showing that the microbiome play an important role in mediating the attraction of gravid sandfly females toward certain types of breeding grounds (20, 21). Even though microbial studies in sandflies are severely neglected and sparse, existing data still indicate that the microbiome play a more prominent role in sandfly biology, ecology, and vectorial potential than previously thought. Understanding the sandfly microbial ecology is crucial as it bears significant implications for the development of novel vector control strategies aimed toward leishmaniasis reduction.

Given the challenges associated with conducting sandfly studies in nature, we designed a laboratory study that evaluated the impact of microbial components, particularly microbiome-rich/depleted nutrition, on sandfly life span and gut microbiome content in relation to the inherited microbiome, thereby contributing to the overall knowledge and understanding of sandfly biology and ecology.

## 2 Method

### 2.1 Sandfly colony

Experiments were conducted on a *Phlebotomus papatasi* colony originating from Sanliurfa, Türkiye (established in 2003). Five-to-seven-days-old females were artificially blood-fed and left to deposit eggs. The eggs were collected, surface sterilized, counted, and transferred to clean pots. All pots were maintained under standard conditions (humidity: 60–70%; temperature: 26 ± 1°C; light:dark photoperiod: 14:10 h), with the variation in food availability. The pots were examined three times per week and all observations were made on three individual replicates. In total, 12 pots with 3,823 eggs were examined during this experiment (Table 1). Larval food was prepared from equal amounts of rabbit chow and faeces (22), and all sandflies were fed with the food obtained from the same preparation batch. Tested rearing pots were divided into three groups. The first test group (T1) was fed on the autoclaved larval food weighing between 0.5–1.5 g depending on the larval stage. The second test group (T2) was overfed, e.g., immature stages of sandflies were provided with high amounts of larval food, ranging from 0.8 g for young larvae to 2.3 g for older larvae. The third test group (T3) was subjected to light starvation conditions where only a limited amount of food was provided. Young larvae were provided with less than 0.4 g, while older larvae were provided with less than 1.2 g per feeding. Control (C) rearing pots were maintained according to the standard insectary protocol and the amount of food and feeding regimen were the same as for the T1 group. Adult sandflies were released from rearing pots three times per week, were provided with 40% sugar solution and were kept under the same conditions as the immature stages.

**Table 1:** Life parameters of sandflies observed under the impact of different nutrition.

	Control group (C)	Group fed with autoclaved food (T1)	Group that was overfed (T2)	Group that was starved (T3)
Initial number of eggs^a^	982	920	949	972
Number of emerged adults	794	573	749	283
Mortality	7.9%	28.2%	9.6%	67%
Developmental period^b^	47 ± 3 days	62 ± 5 days	48 ± 3 days	96 ± 8 days
Longevity of adults	21 ± 2 days	20 ± 2 days	20 ± 3 days	19 ± 2 days

^aThe expressed values show total number of samples from three replicates.^

^bFrom first egg deposition to first adult emergence; Data do not show dissected individuals.^

### 2.2 Monitoring of life parameters

The life cycle of sandflies is very complex and requires a transition through four larval instars and a pupa, to reach the adult stage. Larvae burrow the rearing medium during their development, which makes their precise counting particularly challenging. Hence, eggs were counted at the beginning of the experiment, and the total number of emerged flies was recorded. The mortality rate of immature stages was calculated based on the number of eggs and emerged adults, after considering the number of dissected samples. The number of dead flies was recorded three times per week and the longevity of the flies was noted.

### 2.3 Microbial examination of the sandfly gut content

Individual gut dissections were performed on 360 larvae, 116 pupae, and 120 adults. Due to variations in the speed of larval development among the groups, the timing of dissection differed for each group. Due to the minute size of the larvae, the first dissections were conducted approximately 17–18 days post-hatching for C, T1, and T2, and 25–26 days post-hatching for T3, when the larvae reached a minimum size of 1.8 mm to ensure precise gut dissection. The second dissections were conducted on day 23–24 post-hatching for C and T2, 28–29 for T1, and 40 days post-hatching for T3, with the minimal size of the larvae reaching 2.5 mm. Third larval dissections were conducted when the larvae reached a minimum size of 3 mm (day 28–29 for C and T2, 32 for T1, and 55 days post-hatching for T3). The pupa for dissections were mature (with visible wings, and other adult body characteristics). Unfed adult flies of both sexes were dissected at 48 h post-emergence.

Prior to gut dissection, all samples were surface sterilized in a series of washes (70% ethanol) and rinsed (sterile Phosphate Buffered Saline (PBS)) for 3 cycles. Dissections were performed under the binocular stereomicroscope using sterile equipment and sterile PBS as the dissection medium. The undamaged guts were individually macerated in 100 μL of sterile PBS with a sterile pestle, and 20 μL of the homogenate was immediately plated on Brain Heart Infusion (BHI) (Merck, Germany) agar, Wort Agar (WA) (BioLife Italiana, Italy), and Potato Dextrose Agar (PDA) (Merck, Germany). BHI and WA plates were incubated under aerobic conditions for 24 h at 37°C, and PDA plates were incubated at 26°C for 7 days. Colony Forming Units (CFU) of bacteria and yeast were recorded after 24 h and the total load was calculated. All colonies that displayed morphological differences were further subcultured on BHI/WA until pure cultures were obtained. All agars were prepared according to the manufacturer’s instructions and all plates were checked for potential contaminants by overnight incubation at 37°C.

### 2.4 Microbial examination of the rearing medium

Sampling for the microbial analysis of the medium was conducted on the same days as the sampling for sandfly gut dissections. Per sampling in total 0.4 g of medium was collected from the C, T1, and T2 groups, and 0.2 g from T3. The medium was resuspended in sterile PBS, and serial dilutions were prepared. 20 μL of a diluted medium was plated on BHI, WA, and PDA and incubated as previously noted.

### 2.5 Microbiome identification

**Figure 1:**
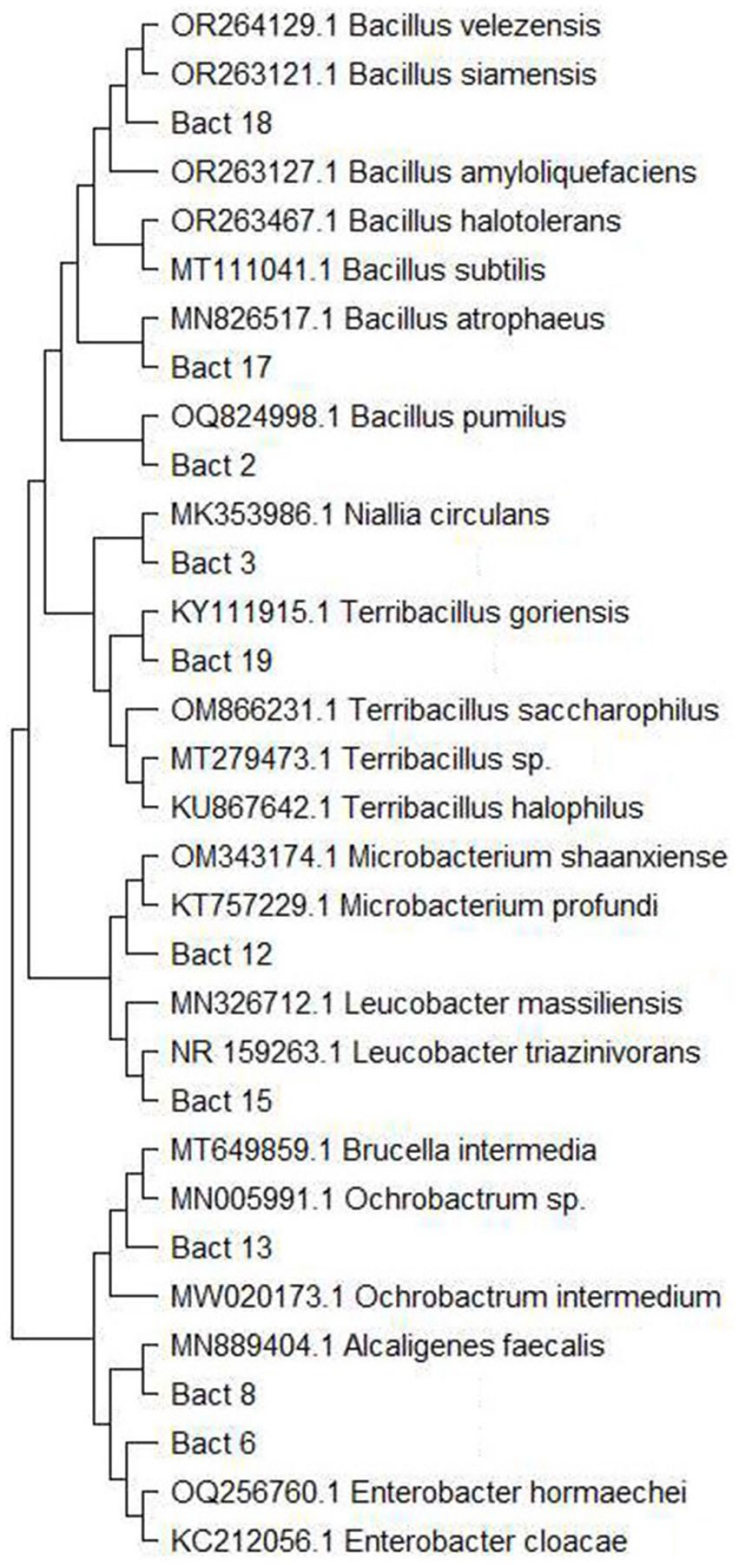
Maximum likelihood phylogenetic tree of bacteria species that were Sanger sequenced for definite species identification. The tree was constructed in MEGA 11 under Tamura-Nei parameter.

Most of the isolated bacteria were identified via traditional methods (23), and only a certain number of isolates that showed similar and/or ambiguous morphological features were subjected to Sanger Sequencing (Figure 1) (14). For this purpose, DNA was extracted from fresh cultures with QIAamp DNA Mini Kit (Qiagen GmbH, Germany), following the manufacturer’s instructions. The extracted DNA was subjected to PCR amplification and subsequent Sanger sequencing with 27F and 1492R primers (24). The obtained sequences were edited in BioEdit (25) and blasted in the NCBI database for homologous sequence search. Considering the problematic taxonomy of bacteria, 98% sequence similarity was considered as a lower threshold at both the genus and species levels, respectively (26). Sequences were aligned using MEGA11 (27), and a maximum likelihood phylogenetic tree was constructed under the Tamura-Nei model (Figure 1). Fungal isolates were identified based on a combination of macro- and microscopic morphology (lactophenol blue staining) (28). The yeasts were identified by morphological characteristics (29).

### 2.6 Statistical analysis

Statistical analysis was performed with Rstudio software (30). The data sets were evaluated for the equality of the two variances using the F-test. One-way and/or two-way analysis of variance (ANOVA) was performed to evaluate the statistical differences between groups. The significance of the differences between group means was evaluated via Student’s T-tests. A *p*-value of 0.05 was used as the threshold.

## 3 Results

### 3.1 Sandfly longevity and life parameters

The developmental parameters of the C group were normal and synchronous, falling inside the regular parameters observed during the long-term colony maintenance, which corresponded to 47 ± 3 days from the first egg deposition to the first adult’s emergence (Table 1). The mortality (7.9%) and adult longevity (21 ± 2 days) rates of the C group also corresponded to the normal parameters. The T1 group that was fed with autoclaved food displayed notable differences in survival rate and developmental length when compared to the C. The mortality of the T1 group amounted to 28.2%, and the developmental time was prolonged (62 ± 5 days; *p*-value: 0.014), while adult longevity was only slightly shorter (20 ± 2 days) (Table 1). Compared with the C group, the T2 group did not display any major differences in the speed of development (48 ± 3 days) or adult longevity (20 ± 3 days), while the mortality rate was slightly higher (9.6%) (Table 1). Test group 3 displayed more irregular and drastically slower (96 ± 8 days; *p*-value: 0.0007) development, as well as strikingly increased mortality (67%), when compared to the C group (Table 1). The longevity of the adult flies in the T3 group was slightly shorter in comparison to the C group (19 ± 2 days; *p*-value: 0.006). Overall, the mortality rate of the larvae was higher, and larval developmental time was longer for sandflies maintained on autoclaved medium (54 ± 3 days), in comparison to the larvae who had plentiful available food with alive and complex microbiome present, i.e., C (38 ± 3 days; *p*-value: 0.004), and T2 (41 ± 3 days; *p*-value: 0.011). The differences in larval development were even more pronounced among sandflies that were exposed to light starvation conditions (85 ± 3 days), in comparison to the larvae from C (*p*-values: 0.0002) and T2 group (*p*-values: 0.0005). This indicates that the presence of live and diverse microbiomes, as well as a sufficient amount of food, plays a role in the optimal development and survival of larvae. No major differences were observed in relation to the duration of the pupal stage of the C, T1, and T2 groups which lasted on average for 7–9 days. Duration of the pupal stage from the T3 group was slightly prolonged (10–11 days) showing statistical significance when compared with C (*p*-value: 0.024) and T2 (*p*-value: 0.025) groups. The adult longevity among the groups was very similar, showing statistical significance only between the control and group exposed to starvation conditions (*p*-value: 0.006) (Table 1).

### 3.2 Microbial content of the rearing medium

**Figure 2:**
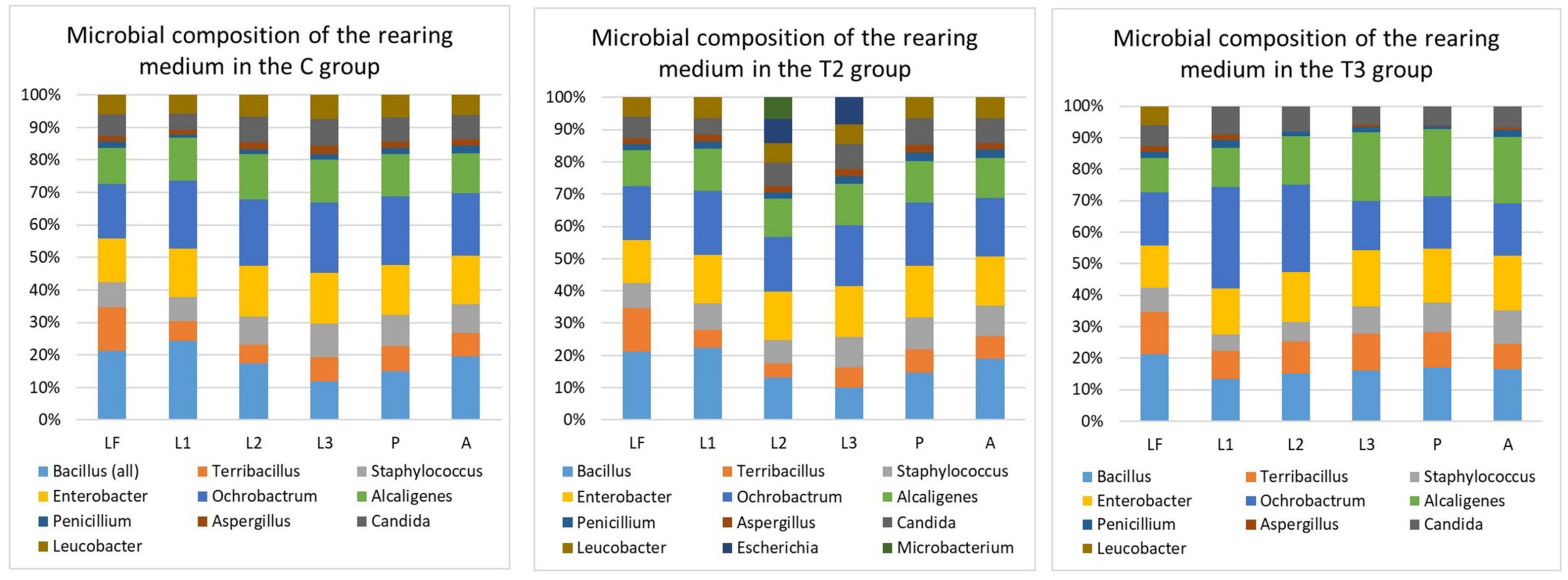
Microbial composition of rearing medium within different groups. T1, Test group 1 was fed with autoclaved medium; T2, Test group 2 was overfed; T3, Test group 3 was exposed to starvation; LF, Larval food; L1, First sampling during larval stage; L2, Second sampling during larval stage; L3, Third sampling during larval stage; P, Sampling during pupal stage; A, Sampling during adult stage. Statistically significant differences within the C group were observed when microbial content of the LF was compared with the microbial content of the rearing medium collected during L1 (*p*-value: 0.0021), L2 (*p*-value: 0.0031), L3 (*p*-value: 0.012), P (*p*-value: 0.018), and A (*p*-value: 0.022). When microbial composition of the rearing medium was compared between L1, L2, L3, P and A sampling, statistically significant differences were seen between L3 and P (*p*-value: 0.0099), L3 and A (*p*-value: 0.027). Within T2 group, statistically significant differences in the microbial content were noted between LF and sampling L1 (*p*-value: 0.015), P (*p*-value: 0.031), and A (*p*-value: 0.0436). There were no statistically significant differences in the microbial composition of the rearing medium between L1, L2, L3, P, and A sampling points. Within T3 group, when compared with microbial content of the LF, statistically significant differences were observed during L1 (*p*-value: 0.00097), L2 (*p*-value: 0.00073), L3 (*p-*value: 0.0011), P (*p*-value: 0.00093), and A (*p*-value: 0.0013). When microbial composition of the rearing medium was compared between L1, L2, L3, P and A sampling, statistically significant differences were seen between L3 and P (*p*-value: 0.033), L3 and A (*p*-value: 0.023). When microbial composition of different sampling points (L1, L2, L3, P, and A) was compared between the groups (C, T2, and T3), statistically significant differences were observed during L1 (C-T2: 0.0014; C-T3: 0.0094; T2-T3: 0.0042), L2 (C-T2: 0.019; C-T3: 0.0013; T2-T3: 0.000033), L3 (C-T3: 0.0029; T2-T3: 0.00055), P (C-T3: 0.0017; T2-T3: 0.00086), and A (C-T3: 0.0021; T2-T3: 0.0013). A *p*-value of 0.05 was used as the threshold during all calculations.

The microbial composition of the larval food was examined at the beginning of the experiment. This microbial composition was used as a baseline with the follow-up detection of several unique records throughout the sampling period within the different examined groups (Figure 2). In the C group, *Bacillus pumilus* (≈ 0.6 × 10^6^) (Figure 1) was recorded only at the second larval sampling (*p*-value: 0.044), and then again during pupal sampling. In the T2 group, *Microbacterium* sp. (≈ 2.4 × 10^6^) was isolated from the second larval sampling, and *Escherichia coli* (≈ 2.8 × 10^6^) was present during the second and third larval sampling. Even with the presence of these unique findings, no statistically significant differences between sampling times were observed within the T2 group (Figure 2). In general, for the C and T2 groups, the overall CFU values of bacteria were lower during the first and second sampling, whereas slightly increased numbers were observed during the third sampling. Contrary to C and T2, within the T3 group we noted the absence of these unique records, with an overall diminished microbial diversity and abundance in the medium (Figure 2). Within the T3 group, *Ochrobactrum intermedium* bacteria were present with higher CFU numbers during the first and second sampling (≈ 4.12 × 10^6^), while its numbers decreased in the third sampling (≈ 2.25 × 10^6^). The CFU numbers of *Alcaligenes faecalis* (the second predominant species within T3 group) were low during the first sampling (≈ 2.75 × 10^6^), and an increase in CFU was observed during the second and third sampling (≈ 3.15 × 10^6^). When compared with the larval food, the microbial composition of the T3 group displayed statistically significant differences during all sampling points (Figure 2). The microbial content of the rearing medium within the T1 group remained unchanged during the experiment, and plates were negative for microbial presence. The medium collected during pupation and 48 h post-emergence of adults showed slightly enriched microbial composition and abundance when compared with the medium collected during the larval period (Figure 2). The microbial increase was mainly reflected in the diversity of species from the *Bacillus* genus. These results show that the presence of actively feeding larvae changes and reduces the diversity and abundance of bacteria present in the rearing substrate (except T1).

Contrary to the relatively diverse bacterial composition, only two species of fungi and one yeast were recorded in the rearing medium. The highest abundance of fungi and yeast was detected in the rearing medium of the T2 group with the species belonging to the genera *Aspergillus, Penicillium,* and *Candida*. The slightly diminished presence of yeasts and the lowest abundance of fungi was observed in T3 group.

### 3.3 Microbial content of the sandfly guts of immature and adult stages

To better understand the composition of the sandfly gut microbiome, we examined the microbial composition of the larval food prior to the experiments and the composition of the rearing medium as larval development progressed. Variations in the microbial composition of the larval gut were observed between all examined groups. However, the microbial content within the same group did not exhibit significant variation with respect to the sampling points (first, second, or third sampling).

**Figure 3:**
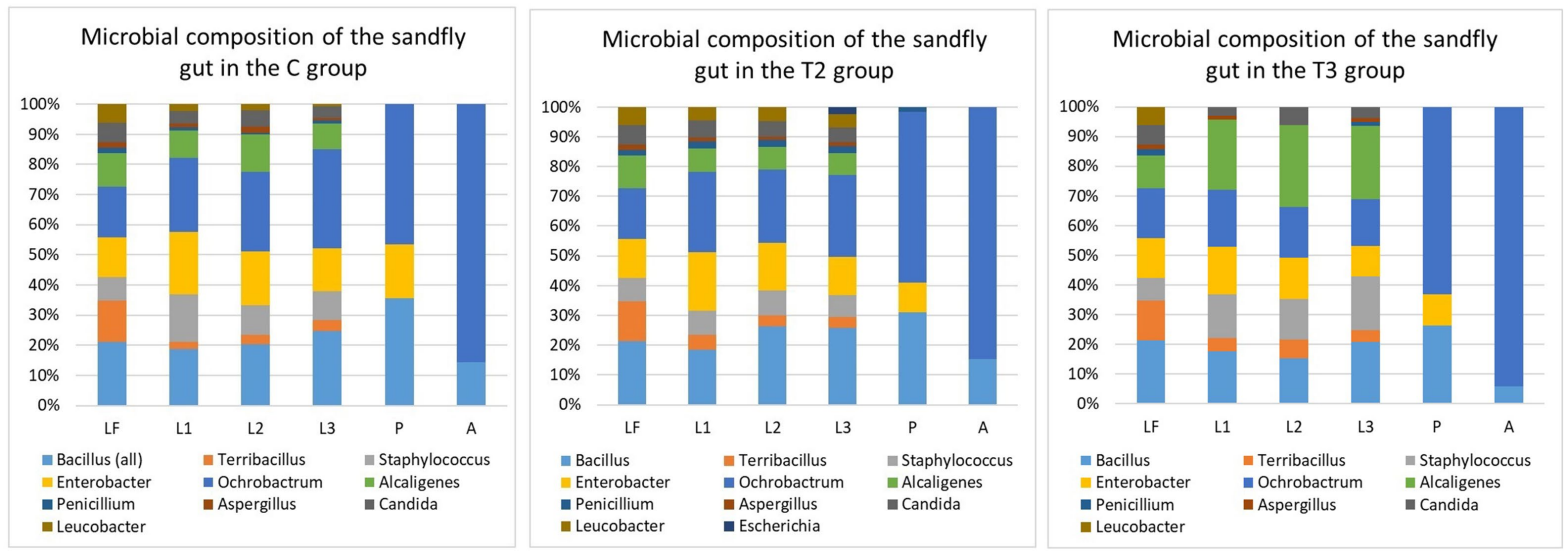
Microbial composition of the sandfly gut within different groups T1. Test group 1 was fed with autoclaved medium; T2, Test group 2 was overfed; T3, Test group 3 was exposed to starvation; LF, Larval food; L1, First sampling during larval stage; L2, Second sampling during larval stage; L3, Third sampling during larval stage; P, Sampling during pupal stage; A, Sampling during adult stage. Statistically significant differences in the microbial composition between the LF and sandfly gut were noted during all sampling points (L1, L2, L3, P, and A) within all evaluated groups (C, T2, and T3) that contained live microbiome. Statistical values within the C group: LF-L1 (*p*-value: 0.00089), LF-L2 (*p*-value: 0.00071), LF-L3 (*p*-value: 0.00075), LF-P (*p-*value: 0.00056), and LF-A (*p*-value: 0.00059). Statistical values within the T2 group: LF-L1 (*p*-value: 0.0016), LF-L2 (*p*-value: 0.0012), LF-L3 (*p*-value: 0.0023), LF-P (*p*-value: 0.0012), and LF-A (*p*-value: 0.0013). Statistical values within the T3 group: LF-L1 (*p*-value: 0.00077), LF-L2 (*p*-value: 0.0008), LF-L3 (*p*-value: 0.0008), LF-P (*p*-value: 0.00067), and LF-A (*p*-value: 0.00066). When microbial composition of different developmental forms within the group was compared, statistically significant differences were noted between L3 and P among all three groups (C group: *p*-value: 0.014; T2 group: *p*-value: 0.0048; T3 group: *p*-value: 0.022). When gut microbial content of different developmental stages was compared between the groups (C, T2 and T3), significant statistical differences during the larval sampling L1 were observed between C and T3 (*p*-value: 0.013), and T2 and T3 (*p*-value: 0.011); the larval sampling L2 between C and T3 (*p*-value: 0.012) and T2 and T3 (*p*-value: 0.013); and the larval sampling L3 between C and T3 (*p*-value: 0.029) and T2 and T3 (*p*-value: 0.012). No statistically significant differences in gut microbiome were noted between C, T2 and T3 groups during pupa and adult stages. When microbial composition of the rearing medium (M) and sandfly gut (G) content was compared, statistically significant differences within C group were noted between ML1 and GL1 (*p*-value: 0.0042), ML2-GL2 (*p*-value: 0.00011), MP-GP (*p*-value: 0.00034), MA-GA (*p*-value: 0.00039). Microbial comparison of M and G within T2 group showed differences between ML1 and GL1 (*p*-value: 0.0026), ML2-GL2 (*p*-value: 0.00036), ML3-GL3 (*p*-value: 0.043), MP-GP (*p*-value: 0.00055), MA-GA (*p*-value: 0.00085). Statistically significant differences in microbial composition between M and G of the T3 group were observed between ML1 and GL1 (*p*-value: 0.013), ML2-GL2 (*p*-value: 0.0077), ML3-GL3 (*p*-value: 0.0025), MP-GP (*p*-value: 0.0022), MA-GA (*p*-value: 0.0025). A *p*-value of 0.05 was used as the threshold during all calculations.

Overall, several species of the *Bacillus*, *Staphylococcus* and *Enterobacter* genus have been commonly found in the larvae gut within all examined groups (except T1) (Figure 3). *Ochrobactrum intermedium* (≈ 1.6 × 10^3^) and *Bacillus subtilis* (≈ 0.9 × 10^3^) were found to be predominant in the sandfly larvae guts of the C and T2 groups, while *Alcaligenes faecalis* (≈ 0.7 × 10^3^) was the most predominant in the guts of the sandflies within the T3 group. *Bacillus pumilus* (≈ 4 × 10^2^) (Figure 1) that was isolated during the second sampling of the rearing medium from the C group, was recorded in the larval guts during the second and third dissections. Interestingly, *Microbacterium* sp. found in the T2 group during the second sampling of the medium was never recorded in the gut of larvae; while *Escherichia coli* (≈ 2.2 × 10^2^) recorded during the second and third sampling of the medium, was recorded in larvae gut only during the third dissection in low number (Figure 3). *Penicillium* was found in larval guts with several isolates from the C group, and predominantly T2, and with only one isolate from T3; while *Candida* was frequently found within the larval gut of the samples from the C and T2 groups, reduced in T3, and absent from T1.

The microbial content of the pupa gut was severely diminished in comparison to the larvae, and only three bacteria (*Ochrobactrum*, *Bacillus,* and *Enterobacter*), and a single isolate of *Penicillium* were detected. With regard to adult flies, only two bacteria demonstrated the potential to survive metamorphosis from the pupa - *Ochrobactrum intermedium* and *Bacillus subtilis*. *Ochrobactrum* (≈ 10^2^) was found in 95% of all dissected adults, while *Bacillus* was found in a notably lower percentage (16%) and with very low numbers (< 20 colonies per gut).

## 4 Discussion

Sandflies have a worldwide distribution, and they occupy a high variety of habitats (13). However, the effects of the microbiome from breeding sites on the biology and ecology of sandflies, as well as the significance of microbes in larval development remains poorly understood. It has been demonstrated that the microbiome plays a significant role in *Leishmania* sp. development in the sandfly gut (17, 18), and that microbes can even affect immune-related gene expression and interaction with *Leishmania* sp. (31); but it is unknown how inherited and/or acquired microbiome impact sandfly life parameters and subsequently their vectorial potential. Although studies on the overall impact of microbiome on sandflies are very scarce, and only a limited number of publications is available, other vector borne insects such as mosquitoes have been explored in greater detail. As seen from the mosquito studies, microbial communities are known to influence mosquito life by modifying essential metabolic and behavioral processes that affect reproduction, development, immunity, digestion, egg survival, and the ability to transmit pathogens (32–38). Given the significant impact of microbes on vital aspects of mosquito biology, this study investigated how microbiome-rich/depleted nutrition, as a key factor in sandfly life, influences their survival, development and longevity with special emphasis on the microbial component. The study showed that even under controlled laboratory conditions the microbiome of both the sandfly gut and rearing medium varied among the examined groups (Figures 2, 3). In general, the presence of actively feeding larvae seems to change and reduce the microbial diversity of the rearing substrate (Figure 2). Microbial recovery was noted in all examined groups (except T1) during the third sampling within a larval stage, as well as during the sampling with predominance of pupa, and 48 h after adults started to emerge. The microbial increase could be attributed to the lower feeding rate, as most of the larvae corresponded to the late 4^th^ instar in addition to the visible presence of multiple pupae and emerging adults. Alternative explanation for bacterial decline and subsequent recovery could be related to larval excretions, which can contain toxic or unfavorable substances that adversely affected the microbiome. Although microbial recovery (increased CFU numbers) was noted among all recorded species of bacteria, diversity increase was noted only among *Bacillus* genera (detection of *Bacillus pumilus* (Figure 1)), which may imply that actively feeding larvae were more attracted to *Bacillus* species. Studies conducted on adult sandflies showed that gravid females are attracted toward certain bacteria (21, 39, 40), but it was not evaluated whether larvae are guided by the same/similar attraction. It is still unclear if specific bacteria, such as species from the *Bacillus* genus, were more attractive to the larvae, or if their consumption was determined by chance. It would be recommended that further studies examine if sandfly larvae are attracted by specific microbes, as this knowledge could aid in developing biological control strategies at the larval stage.

The presence/absence of a live microbiome plays a role in sandfly development, and it can have both positive and negative effect. From C group can be observed that optimal amount of food, containing live and diverse microbiomes plays a crucial role in sandfly development and survival. Conversely, the absence of a live microbiome as seen in the T1 group, can adversely affect the sandflies by prolonging the development of immature stages and increasing their mortality. Similar observations pertaining to the impact of the microbiome on the survival and development of the immature stages were noted in mosquitoes. Reports document that a diverse microbiome from the habitat is necessary for growth and molting, and that mosquito larvae exhibit higher mortality and/or delayed development all up to the pupal stage when the abundance of microbes in the habitat was reduced (41–44).

Moreover, as seen from T2 group, too many microbes can negatively affect immature stages of sandflies. It is suggested that microbial, especially fungal overgrowth can lead to higher mortality of larvae, as larvae may entangle in fungal hyphae (22). In this study, overgrowth was a direct consequence of overfeeding, as substantial quantities of uneaten food were exposed to hot and humid conditions within the rearing pots, which are perfect for microbial growth. Although fungal overgrowth was cleaned regularly, it is possible that hyphae presence affected young larvae leading to their death, therefore resulting in higher mortality within the T2 group.

While the presence of microbiome and its composition play a role in sandfly development, another deciding factor is the amount of available food. The T3 group that was exposed to the light starvation conditions displayed a notably higher mortality rate and prolonged development. Even though approximately 96% of the eggs hatched, by the end of the 4^th^ instar only a fraction of the larvae was alive. The mortality of larvae seemed to be highest within the first 2 weeks after hatching, and larval number continued to decline throughout the active phases of feeding. It is suspected that the presence of a live microbiome contributed to the overall survival of the larvae, and that the mortality rate would be even higher if the T3 group was fed with microbiome-depleted food. Although specific body measurements were not taken, it is authors’ observation that all larvae that had enough food available (C, T1 and T2), regardless of the fact that microbial flora was viable or not, displayed marginally larger body size in comparison to T3 group that was exposed to light starvation conditions. The larvae from the T3 group seemed slender and smaller, demonstrated decreased activity, and their digestive tracts were nearly devoid of content. Larvae, as the actively feeding stage of immature development are more affected by nutrition status. Hence, the prolonged duration of the immature development was mostly reflected in the larval instars, with similar findings being reported in mosquitoes (45). Even though the changes in the duration of the pupa and adult longevity were rather minor, they were most likely caused by the developmental conditions of the larvae. Nutrition does not play a major role in the process of pupation, as pupae do not feed; but preservation of the pupal gut microbiome is important as most of the microbes are eliminated during the histolysis of the digestive tract tissue during metamorphosis (46) (Figure 3). Among all groups that were fed with food containing live microbiome (C, T2, and T3 groups), it was observed that females dissected 48 h post-emergence displayed the presence of alive microbiome in their guts (Figure 3). The diversity and abundance of inherited bacterial communities were rather limited, with *Ochrobactrum* being found at a higher percent (95%) compared to *Bacillus* (16%). Previous studies demonstrated that the low presence of inherited microbiomes can recover through CFU increase (14), indicating that they play a role in the adult sandfly life functions and potential *Leishmania* susceptibility.

As all adults were exposed to the same conditions and food source, no major differences in adult longevity were observed between C, T1, and T2 groups. The T3 group, in comparison with the C group displayed a slightly shorter longevity, which could point toward the role of nutrition and microbial component in the sandfly fitness. The authors’ observation is that sandflies from the T3 group were marginally smaller, which may be a direct consequence of previous observations made on the larval stage, wherein smaller larvae lead to smaller pupae, resulting in smaller adults.

Given that this experiment was conducted under laboratory conditions, with the food source whose microbial composition was limited and controlled, it was still possible to isolate and identify two bacterial communities of the inherited microbiome. It is highly likely that the diversity of sandfly inherited microbiome found in nature is much greater. The investigations of the inherited microbiome and its cultivation can contribute to the development of leishmaniasis control methods, in the same manner as the utilization of mosquito-derived microbes contributed to mosquito pathogen control (47).

In conclusion, sandflies contribute to the microbial shifts of the rearing medium under laboratory conditions, and the same can be expected in nature. Concurrently, the medium composition impacts the diversity and abundance of microbiomes present in the gut of immature stages and promotes sandfly development and survival. The diversity of microbial flora in the medium impacts the composition of the inherited microbiome, which may influence sandfly susceptibility to *Leishmania*, vector capacity and even severity of disease manifestation.

## Data Availability

The original data are available in a publicly accessible repository of the GenBank under the accession numbers: PQ784271-PQ784280.
